# The Activity of Prebiotics and Probiotics in Hepatogastrointestinal Disorders and Diseases Associated with Metabolic Syndrome

**DOI:** 10.3390/ijms23137229

**Published:** 2022-06-29

**Authors:** Alicia Rodríguez-Pastén, Nury Pérez-Hernández, Javier Añorve-Morga, Rubén Jiménez-Alvarado, Raquel Cariño-Cortés, Teresa Sosa-Lozada, Eduardo Fernández-Martínez

**Affiliations:** 1 Department of Medicine, Health Sciences School, Autonomous University of Hidalgo State, Pachuca 42090, Mexico; alicia.r.pasten@gmail.com (A.R.-P.); raquelcarcortes@gmail.com (R.C.-C.); maria_sosa@uaeh.edu.mx (T.S.-L.); 2 Programa Institucional de Biomedicina Molecular, Escuela Nacional de Medicina y Homeopatía, Instituto Politécnico Nacional, Mexico City 07320, Mexico; nperezh@ipn.mx; 3 Área Académica de Química, Instituto de Ciencias Básicas e Ingeniería, Universidad Autónoma del Estado de Hidalgo, Mineral de la Reforma 42184, Mexico; anorvej@uaeh.edu.mx; 4 Instituto de Ciencias Agropecuarias, Universidad Autónoma del Estado de Hidalgo, Av. Universidad km. 1, Tulancingo 43600, Mexico; ruben_jimenez@uaeh.edu.mx; 5 Laboratory of Medicinal Chemistry and Pharmacology, Centro de Investigación en Biología de la Reproducción, Área Académica de Medicina, Instituto de Ciencias de la Salud, Universidad Autónoma del Estado de Hidalgo, Pachuca 42090, Mexico

**Keywords:** gut microbiota, prebiotics, probiotics, metabolic syndrome, liver, lipids, immunomodulation

## Abstract

The components of metabolic syndrome (MetS) and hepatogastrointestinal diseases are widespread worldwide, since many factors associated with lifestyle and diet influence their development and correlation. Due to these growing health problems, it is necessary to search for effective alternatives for prevention or adjuvants in treating them. The positive impact of regulated microbiota on health is known; however, states of dysbiosis are closely related to the development of the conditions mentioned above. Therefore, the role of prebiotics, probiotics, or symbiotic complexes has been extensively evaluated; the results are favorable, showing that they play a crucial role in the regulation of the immune system, the metabolism of carbohydrates and lipids, and the biotransformation of bile acids, as well as the modulation of their central receptors FXR and TGR-5, which also have essential immunomodulatory and metabolic activities. It has also been observed that they can benefit the host by displacing pathogenic species, improving the dysbiosis state in MetS. Current studies have reported that paraprobiotics (dead or inactive probiotics) or postbiotics (metabolites generated by active probiotics) also benefit hepatogastrointestinal health.

## 1. Introduction

Current lifestyle patterns and unhealthy eating habits may lead to various diseases, such as gastrointestinal and mainly metabolic disorders. Metabolic syndrome (MetS) is a combination of risk factors, including an imbalance in carbohydrates and lipid metabolism, and can be manifested as obesity, diabetes (or hyperglycemia and insulin resistance), hyperlipidemia (elevated triglyceride (TG) and cholesterol (COL) levels), cardiovascular disease (atherosclerosis and hypertension), and even non-alcoholic fatty liver disease (NAFLD) [1,2,3,4]. On its own, NAFLD is known as the hepatic manifestation of MetS. It is considered the most common chronic liver disease worldwide and is defined as the accumulation of ≥5% fat in the liver. Non-alcoholic steatohepatitis (NASH) is a progressive disease that includes chronic inflammation, and it is estimated that up to 20% will develop cirrhosis that can progress to liver failure and even hepatocellular carcinoma (HCC) [5,6,7].

Interest in the importance of the intestinal microbiota has increased because several studies have shown its benefits to the host. The term microbiota refers to the population of microorganisms that colonize a particular site in the body. These microorganisms offer many benefits to the host since they contribute to the metabolism of nutrients and drugs, prevent colonization by pathogenic microorganisms, maintain intestinal barrier function and regulate the immune system [8,9]. There may be an imbalance in the microbiota due to various factors—diet, habits, various diseases, and the consumption of medications such as antibiotics; such alterations are associated with the development of chronic diseases, for example, the previously mentioned metabolic disorders and gastrointestinal diseases, such as inflammatory bowel disease, colitis and even gastritis caused by *Helicobacter pylori* [10,11,12,13,14]. The consumption of probiotics and prebiotics in food or supplements is usually suggested to reshape the microbiome. To be considered probiotics, non-pathogenic microorganisms must survive through the gastrointestinal transit conditions and provide health benefits to the host. Most of them mainly belong to the family of lactic acid bacteria (LAB), specifically the genera *Lactobacillus* and *Bifidobacterium*, and some species of the genera *Streptococcus* and *Enterococcus*. Certain bacteria are considered probiotics, but some species of yeasts have also been classified in this group; among them are *Saccharomyces* spp. The molecules that stimulate the growth of probiotic populations and their activities are considered prebiotics [11,12,15,16]. All the scientific genera and species in this Review were updated and validated, see Appendix A.

In general, foods with prebiotic and probiotic properties have been around for several centuries and have been used by various ancestral cultures as an alternative mainly for treating gastrointestinal diseases. Currently, food products or supplements with these properties are not fully regulated. Therefore, this has increased interest in developing various pre-clinical and clinical studies that support the impact and benefits attributed to the consumption of prebiotics and probiotics [11]. The aim here is to review the numerous studies which have provided evidence of the importance and role of probiotics and prebiotics in hepatogastrointestinal diseases related to MetS, their effects on the immune system, and the proposed mechanisms of action.

## 2. Prebiotics, Probiotics, Synbiotics, Paraprobiotics and Postbiotics

Prebiotics has been defined as “indigestible food ingredients that beneficially affect the host by selectively stimulating the growth and/or activity of bacteria”; however, in 2016, the definition was modified by the International Scientific Association of Probiotics and Prebiotics (ISAPP) as “a substrate that is selectively used by host microorganisms that confer health benefits” [11,17]. Other definitions proposed are “indigestible compounds that, through their metabolization by microorganisms in the intestine, modulate the composition and/or activity of the intestinal microbiota, thus conferring a beneficial physiological effect on the host” [18]; however, all definitions focus on compounds that contribute to the growth of probiotics. Prebiotics can be classified as fibers; prebiotic compounds include oligosaccharides, fructooligosaccharides (FOS), galactooligosaccharides (GOS), β-glucans, and inulin. These selectively stimulate the proliferation of Lactobacillus spp. and Bifidobacterium spp., attenuate the growth of pathogenic species, decrease intestinal pH, are resistant to hydrolysis and gastrointestinal enzymes, and are not absorbed in the upper gastrointestinal tract. Hence, prebiotics provide a medium for beneficial microorganisms [17,19,20]. On the other hand, the microbiome can be defined as a set of complex communities of diverse microorganisms, such as bacteria, fungi, viruses, and protozoa, that colonize various parts of the organism [21]. The term microbiome includes certain species that contribute to host health, called probiotics; these offer numerous benefits, contributing to gastrointestinal health, metabolic processes, and immune system regulation [9].

Probiotics have also undergone various modifications; they are currently defined as “living microorganisms which, when administered in adequate quantities, confer a health benefit to the host”; ISAPP has proposed keeping this definition. To consider a microorganism as a probiotic, it must meet various criteria, among which are: survival in the conditions of the gastrointestinal tract (resistance to acidic pH, bile salts, gastric juice), adherence to the intestinal epithelium, and safety for use or consumption (non-pathogenic microorganisms) [22,23]. Currently, two dominant phyla are known, which make up 90% of species in the human digestive tract, Bacteroidetes and Firmicutes [24]. The combination of prebiotics and probiotics is called synbiotics. The use of synbiotics can synergize the beneficial effect on the host; therefore, this combination has shown promise in the treatment and prevention of metabolic and gastrointestinal disorders [20]. 

The term paraprobiotic has been proposed to refer to dead probiotics, inactive cells, or cell fractions. Specifically, “non-viable cells (intact or not) or cell extracts that conferred health to the host when administered in adequate amounts” have been shown to benefit health, for example, by inhibiting the adhesion of pathogenic bacteria, and have shown positive immune responses. Moreover, unlike probiotics, paraprobiotics do not lose bioactivity when coadministered with antibiotics or antifungal agents. Therefore, they can be helpful in people with reduced immunity, altered intestinal barriers, or sepsis, and in premature babies [25,26,27]. Similarly, it has been proposed that probiotics can produce postbiotics. Postbiotics have been defined as cell-free, biogenic supernatants, metabolites, and metabolic waste products resulting from probiotic activity. They have also been defined to include any effect obtained from the metabolites of probiotics or any molecule extracted or secreted that directly or indirectly offers health benefits to the host. These include enzymes, exo- and endopolysaccharides, surface proteins, vitamins, organic acids, fatty acids, and peptides [25,28].

## 3. Dysbiosis

Several factors impact the microbiota composition, mainly diet, drugs, and the immune system in general. As a result of variations in microbiota composition, a state of dysbiosis may occur [29]. Dysbiosis (also known as dysbacteriosis) is usually defined as “a compositional and functional alteration in the microbiota determined by a set of environmental and host-related factors that disturb the microbial ecosystem to the extent that exceeds its resilience and resilience capacities” [21]. Furthermore, an imbalance between the abundance of beneficial organisms and pathogens can contribute to the development of diseases, although it can also result from them. Dysbiosis presents some typical characteristics, among which an increase in the proliferation of pathogenic bacteria, a significant decrease or even the disappearance of commensal species, and a general loss of diversity in the microbiota from the first years of the life of an individual stand out. This imbalance is associated with gut barrier dysfunction and bacterial translocation; it is also accompanied by gastrointestinal symptoms, such as inflammatory bowel disease and irritable bowel syndrome, obesity, diabetes, metabolic disease, cardiovascular disease, immune system disorders, and chronic liver diseases [30,31,32,33].

Diet influences microbiota imbalance, especially a diet low in fiber and the consumption of dietary xenobiotics. Genetic and environmental factors are also involved in the colonization of specific taxa. Other factors linked to dysbiosis are circadian alterations and the consumption of drugs, mainly antibiotics and nonsteroidal anti-inflammatory drugs [21,29]. 

## 4. Prebiotic, Probiotic and Synbiotic Activities in Hepatogastrointestinal System Diseases

Many studies have supported the beneficial role of probiotics and prebiotics in diseases of hepatogastrointestinal origin since their functionality in preventing and improving these diseases has been verified [12,23,34,35].

### 4.1. Liver Diseases

Bile acids (BAs) are essential factors in the axis between the liver and the gut; the gut microbiota carries out biotransformation of primary to secondary BAs and is implicated in the progression of chronic liver disease. Prebiotics, probiotics, and synbiotics have improved some liver diseases significantly and have been used to modulate microbiota in patients with liver diseases [30,36]. Prebiotics such as sapogenins, derived from kammogenin, manogenin, gentrogenin, and hecogenin, decrease the expression of tumor necrosis factor (TNF)-α and the accumulation of hepatic lipids by contributing to the regulation of the expression of lipogenic genes, such as the sterol regulatory element-binding protein 1c (SREBP-1c), and increase the expression of genes involved in lipid oxidation, such as the peroxisome proliferator-activated receptor (PPAR)-α, in addition to increasing the population of the species *Akkermansia muciniphila* in an experimental model of obesity and hepatic steatosis, induced with a high-fat diet [34]. Consumption of polysaccharides decreased serum levels of liver damage markers, such as aspartate aminotransferase (AST), alanine aminotransferase (ALT), malondialdehyde (MDA), and the cytokines transforming growth factor (TGF)-β, TNF-α, interleukin (IL)-1β, and IL-6, and increased IL-10 and endogenous antioxidants, such as superoxide dismutase (SOD) and glutathione peroxidase (GSH-Px). In addition, mice treated with prebiotics exhibited improvement in intestinal permeability and decreased plasma lipopolysaccharide (LPS) levels in cirrhosis [30,37,38].

There are reports about the aggravation of diseases, such as NAFLD (which may advance to NASH, fibrosis, and even cirrhosis), alcoholic steatohepatitis, cholangitis, hepatic encephalopathy, and hepatocarcinoma, related to dysbiosis states [24,39]. It has been reported that in the mentioned diseases, changes occur in the intestinal microbiota, which leads to an intensification of intestinal permeability and alterations to the immune system and the inflammatory response. Therefore, some species of probiotics, such as *Lacticaseibacillus casei*, help reduce bacterial translocation, while *Leuconostoc mesenteroides, Lacticaseibacillus* *paracasei, Lactiplantibacillus* *plantarum*, and *Pediococcus pentosaceus* contribute to diminution in populations of certain pathogens, such as *Escherichia coli,* in addition to decreasing blood ammonia levels and reducing bacterial infections in liver transplant patients [12,23,40]. There is also evidence that a change in diet by increasing the consumption of dietary fiber can regulate the intestinal microbiota (increasing the Bacteroidetes/Firmicutes ratio), in addition to preventing liver fibrosis and reducing serum levels of proinflammatory cytokines (TNF-α, IL-β, and IL-6) while increasing IL-10 and IFN-γ [41]. *Lactobacillus delbrueckii* subsp. *bulgaricus* and *Streptococcus salivarius* subsp. *thermophilus* reduce liver aminotransferases and inflammatory markers in NAFLD patients, who are at increased risk of developing HCC, even without liver cirrhosis; reductions in levels of these markers could improve intestinal permeability and translocation of LPS, thus preventing the development of NAFLD and/or progression to NASH [39]. *Lactobacillus* species have shown beneficial effects in NAFLD/NASH prevention. For example, *Lacticaseibacillus* *paracasei, L. rhamnosus Lactiplantibacillus plantarum,* *Lactobacillus* *acidophilus,* and *Bifidobacterium* (themselves or in the synbiotic complex) are associated with significant decreases in NF-κB, hepatic fat accumulation, and steatosis, as well as lower ALT, AST, TNF-α, IL-4, and serum lipids levels in pre-clinical and clinical studies; in addition, oligofructose and lactulose can modulate the metabolism and regulate Gram-positive and Gram-negative bacterial populations [42,43]. 

### 4.2. Colitis

Inflammatory bowel diseases (IBD), including ulcerative colitis (UC) and Crohn’s disease, are characterized by chronic inflammation of the intestinal mucosa. This results in an imbalance of the immune system and the predominance of proinflammatory processes over anti-inflammatory ones [14]. Probiotics, prebiotics, or synbiotics have been shown to result in significant reductions in markers of inflammation, such as C-reactive protein (CRP), TNF-α, I1-α, IL-6, IL-8, and IL-10 [44,45]. Several probiotic strains have been studied and have shown significant benefits, namely, *E. coli Nissle* 1917, *Lacticaseibacillus* *rhamnosus,* Bifidobacteria, *Saccharomyces boulardii*, probiotic complex VSL#3, and synbiotic complex (*Bifidobacterium longum* and inulin). These have been used as maintenance therapy in patients with colitis, showing promising results for clinical remission; furthermore, in animal models, it has been observed that they lead to the inhibition of NF-κB, IL-1β, and TNF-α expression through the TLR4-NF-κB signaling pathway. As a result, the expression of proinflammatory cytokines and TLR is reduced, and the level of regulatory cytokines increases [14,45]. Some authors have evaluated the effect of probiotics in experimental models of colitis. *Lactobacillus* spp. are considered probiotics; indeed, the species *Lactiplantibacillus plantarum*, *Lacticaseibacillus* *paracasei*, *Levilactobacillus brevis*, and *Agrilactobacillus composti* have shown anti-inflammatory effects by decreasing IL-8 and IL-22, besides improving intestinal hyperpermeability in a model of dinitrobenzene sulfonic acid-induced colitis. Furthermore, *Ligilactobacillus salivarius* and *Bifidobacterium longum* subsp. *infantis* mitigate an IL-10 KO model by reducing IFN-γ, TNF-α, and IL-12, while maintaining the production of TGF-β. *Faecalibacterium prausnitzii* has been shown to block the activation of the nuclear factor NF-κB and the production of IL-8 [35,46]. In animal models, it was observed that *Limosilactobacillus reuteri* prevents the expansion of colitis; in addition, inflammation, mucosal damage, and damage seen on histologic analysis are reduced by orally administered inulin [47].

Synergy 1 synbiotic complex (inulin and FOS) and *Bifidobacterium longum* significantly decreased serum CRP and beta-defensins 2, 3, 4, TNF-α, and IL-1α in patients with ulcerative colitis. In vitro studies potentially demonstrate beneficial immunomodulatory effects of paraprobiotics, such as the regulation of the expression of TNF-α, IL-12, and IL-8, and the nuclear translocation of NFκB; therefore, paraprobiotics have been shown to possess anti-inflammatory, immunomodulatory, antiproliferative, and antioxidant properties [48,49]. 

### 4.3. Gastritis

The reduced diversity of the gastric microbiota has been related to mucosal lesions, such as gastric ulcers, polyps, and gastritis [10]. *Helicobacter pylori* is a Gram-negative bacterium that provokes common infections related to gastritis and peptic ulcer disease. Probiotics, mainly *Lactobacillus* species, increase *H. pylori* eradication rates. Hence, antagonizing *H. pylori* through the regulation of anti-inflammatory cytokines reduces gastric acidity, stimulates mucin secretion, or inhibits *H. pylori* adhesion, suppressing their growth by secreting antibacterial substances, such as lactic acid, short-chain fatty acids (SCFA), H_2_O_2_, and bacteriocins [13,50]. *Saccharomyces boulardii* was evaluated in some studies and has decreased the risk of diarrhea in patients receiving triple eradication therapy for *H. pylori*; it has been observed to induce morphologic changes and cellular damage in the pathogen, reducing its populations [51]. 

Various reports suggest that *Streptococcus salivarius* subsp. thermophilus, *Ligilactobacillus salivarius*, *Limosilactobacillus reuteri*, *Lactobacillus acidophilus*, and *Lactiplantibacillus plantarum* can reduce *H. pylori* colonization and secretion of IL-8, NF-κB activation, inhibit Smad7 expression, weaken the gastric mucosal inflammatory response, and, when applied before infection, reduce the degree of gastritis. Probiotic strain Leptospira johnsonii No. 1088 could suppress *H. pylori* in in vitro and in vivo models; the same heat-killed strain showed antibacterial effects. The suggested mechanisms occur through competitive inhibition, improvement of mucosal barriers, regulation of immunity, and the secretion of antibacterial metabolites; for example, the postbiotics lacticin A164 and BH5 secreted by Lactococcus lactis, reuterin by Limosilactobacillus reuteri, and bacteriocins by Bifidobacterium strains showed significant efficacy against *H. pylori* [52,53,54]. These data are summarized in the Table 1.

## 5. Prebiotics and Probiotics in Disorders Associated with Metabolic Syndrome

Metabolic disorders originate from several factors, although they are often related to lifestyle and diet; several studies address the role that the microbiota plays in the course of these disorders since the beneficial effects on their prevention and treatment are known (Table 1) [69,75]. Various species of probiotics, such as *Lactiplantibacillus plantarum*, *Lacticaseibacillus paracasei, L. rhamnosus, Lactobacillus acidophilus, L. gasseri,* and *Bifidobacterium longum*, have favorable effects, such as the upregulation of genes involved in the transport and metabolism of carbohydrates, improvement in metabolic pathways (oxidation) of lipids, nutrient absorption, and intestinal digestion, as well as the modulation of BA metabolism (favoring enterohepatic recirculation of BAs), decrease in plasma glucose, cholesterol, lipoproteins, TG, hepatic triacylglycerols, and renal lipids, and the deconjugation of bile salts and loss of body weight (reduction in adipose tissue mass) [75,76,77]. Some effects of sapogenins include increasing glucose tolerance, expression of genes involved in lipid oxidation, decreasing weight and body fat, and controlling glucose, TG, and cholesterol in the blood [34]. 

### 5.1. Obesity

Obesity is abnormal or excessive fat accumulation with low-grade chronic systemic inflammation, resulting from genetic and lifestyle factors, including energy imbalance between consumed and expended calories and changes in eating habits that can increase fat intake. Obesity is associated with metabolic diseases, including diabetes, cardiovascular disease, hyperlipidemia, and NAFLD, which are characteristics of MetS; the main consequence of a high-fat diet is the impaired action of insulin and the regulatory mechanisms of body weight [78,79,80].

Some prebiotics (oligofructose) and probiotics, including Bifidobacterium (*Bifidobacterium* *breve B3*, *B. longum* and *B. infantis*) and Lactobacillus (*Lacticaseibacillus casei strain Shirota*, *L. rhamnosus*, *Lactobacillus gasseri* and *Lactiplantibacillus plantarum*) have been used in patients and obese animal models, showing lowered triglyceride levels and adipose tissue mass, waist circumference reduction, and suppression of weight gain, fat depots, and white adipose tissue [79]. Food intake with FOS, GOS, oligofructose, inulin, and polyunsaturated fatty acids regulate total cholesterol (TC), LDL-C, and CRP levels, exhibit anti-obesity effects, and reduce body weight and body fat and IL-6 serum levels in overweight or obese patients; even synbiotics (GOS + FOS + Bifidobacterium breve) have prevented diet-induced obesity in adult mice when administered in early life [81]. Probiotic strains such as *Lactiplantibacillus plantarum* can improve lipid metabolism by downregulating the accumulation of lipids and regulating fat mass by influencing adipogenesis in maturing pre-adipocytes, and the anti-obesity effects of other species, such as *Lactobacillus gasseri*, *Lacticaseibacillus* *casei*, *L. rhamnosus* and *Bifidobacterium* *longum*, are known [82]. Probiotic strains *Lacticaseibacillus casei*, *strain Shirota*, *L. rhamnosus*, *Lactobacillus gasseri* and *Lactiplantibacillus plantarum*, as well as *Bacillus infantis*, *Bifidobacterium* *longum* and *B. breve*, have been widely used in animal models of obesity. Studies reported that these species showed less weight gain, fat accumulation, and white adipose tissue. Other species, such as *Pediococcus pentosaceus*, decreased body weight gain, visceral fat, and liver lipid content in high-fat diet (HFD)-induced obese mice; prebiotics FOS also had beneficial effects on plasma lipid levels. The anti-obesity effects can be associated with the downregulation of genes involved in lipid metabolism, the upregulation of PPAR-α and PPAR-γ, and the downregulation of SREBP-1c [80]. Akkermansia muciniphila colonizes the mucosal layer in the intestines and modulates basal metabolism. It has been evaluated in animal models and human trials, showing a positive effect on obesity because it enhances the organism’s metabolism and has considerable potential for treating obesity-related metabolism [2].

Synbiotics cause beneficial effects on weight reduction via their involvement in gut microbiota modulation and the production of metabolites or postbiotics (for example, SCFA) by inhibiting lipogenesis through the hepatic fatty acid synthase downregulation. Specifically, *Lactobacillus gasseri* combined with galactomannan and/or inulin fibers exerted weight reduction and anti-inflammatory properties; the anti-obesity effects may be due to the synergism for SCFA production and microbiota regulation [83]. The beneficial effects of postbiotics have been observed on body weight, insulin sensitivity, and glucose balance; SCFA can also improve the intestinal barrier, reduce inflammation, positively affect lipid metabolism, and protect against obesity through a high-calorie diet [84].

### 5.2. Atherosclerosis

Obesity, diabetes, and metabolic syndrome, including hyperlipidemia, are the most common factors contributing to an increased risk of developing cardiovascular disease. IFN-γ, IL-12, IL-6, TGF-β, and TNF-α are evident in atherosclerotic plaques [85]. Dysbiosis and intestinal permeability are associated with inflammation and subsequently the translocation of LPS into the blood, playing a vital role in the modulation of Toll-like receptors and their downstream targets; indeed, some metabolites are related to atherosclerosis, mainly SCFA, trimethylamine N-oxide (TMAO), and secondary BAs [86]. It has been observed that the composition of the gut microbiota determines TMAO levels because microorganisms show different abilities to produce TMAO; for example, *Prevotella* has a higher capacity for synthesizing TMAO than *Bacteroides* [87]. Several mechanisms for the role of TMAO in atherosclerosis and thrombosis have been proposed, including the effects of TMAO on inflammation, cholesterol metabolism, and transport. It has been observed that TMAO increases the production of pro-inflammatory cytokines, such as TNF-α and IL-1β, but decreases IL-10 and is related to dyslipidemia and inflammation. On the other hand, BAs can regulate lipid metabolism by binding with farnesoid X receptor (FXR) and Takeda G-protein receptor-5 (TGR-5) receptors [88,89]. Other studies suggest that prebiotics (or synbiotics), including FOS, GOS, and inulin, can modulate the growth of beneficial microbiota. These approaches help to modulate bacteria to transform precursors into trimethylamine (TMA) and improve the bacterial ability to deplete it or help to modulate bacteria devoid of the genes responsible for converting carnitine or choline to TMA; for example, Methanobacteria has been shown to deplete both TMA and TMAO [90].

High levels of blood cholesterol (hypercholesterolemia) constitute a significant risk factor for the development of atherosclerosis and even stroke. The reduction of TC and LDL-C concentrations in the blood significantly decreases the risk of cardiovascular diseases; therefore, gut microbiota regulation of blood lipids, especially cholesterol, through their role in BA biotransformation and the generation of postbiotics, is vital, as mentioned above [91]. 

Oxidative stress is known to play a role in the course of cardiovascular disease. Lactobacillus and Bifidobacterium genera prebiotics, such as *B. animalis* 01, can scavenge hydroxyl radicals and superoxide anions; *Lactiplantibacillus plantarum*, *Lacticaseibacillus rhamnosus,* and inulin protect against oxidative stress. Several studies have reported that *Streptococcus salivarius* subsp. *thermophilus*, *Lactobacillus delbrueckii* subsp. *bulgaricus*, *Lactobacillus acidophilus*, and *Bifidobacterium animalis* protect against atherosclerosis by significant reduction in TC, LDL-C, and TG serum concentrations [92,93,94]. 

### 5.3. Diabetes Mellitus

Diabetes mellitus is a chronic disease characterized by elevated blood glucose levels originating from autoimmune β-cell destruction (type 1 diabetes) or a progressive loss of pancreatic function due to inadequate insulin secretion by β-cells as a consequence of insulin resistance exerted by peripheral tissues, such as the liver, muscle, and adipose tissue (type 2 diabetes). Prebiotics, probiotics, and postbiotics (such as SCFA) have beneficial effects on this metabolic disorder by anti-inflammatory and hypoglycemic effects and insulin regulation [95]. *Bifidobacteria* and *Akkermansia muciniphila* are found in small amounts, whereas *Enterococci, Escherichia coli*, *Lactobacillus gasseri*, *Streptococcus mutans*, *Proteobacteria*, *Clostridiales,* and Firmicutes are significantly higher; the metabolites produced by these bacteria are related to deleterious metabolic effects on glucose homeostasis and insulin resistance in type 2 diabetes [1]. *Levilactobacillus brevis*, *Streptococcus salivarius* subsp. *thermophilus*, *Bifidobacterium,* and other species of *Lactobacillus* are beneficial in type 1 diabetes because they reduce blood glucose levels via gamma-aminobutyric acid (GABA), NF-κB, inhibit IL-1β, prevent TNF-α upregulation, and stimulate IL-10, thereby preserving the functioning of β-cells and reducing serum α-amylase action, and favoring glycemic index mechanisms by restricting carbohydrate absorption and hydrolysis [96].

Some authors report that animal studies with mice, combining probiotics and/or prebiotics with antidiabetic medications showed an improvement in glycemic control and insulin sensitivity; for example, *Bacteroides fragilis*, *Akkermansia muciniphila*, *Lactiplantibacillus plantarum*, and *Lacticaseibacillus casei* induce IL-10, which has been shown to improve both insulin resistance and glucose metabolism [97]. Oral probiotic administration to type 1 diabetic mice stimulates the production of IL-10. There is also evidence of the role of probiotics in cases of type 2 diabetes. The anti-inflammatory effects of *Akkermansia muciniphila*, *Bifidobacteria*, and *Lactobacilli* protect against type 2 diabetes by modulating gut barrier integrity and preventing bacterial translocation associated with bacteria butyrate synthesis or conjugated α-linolenic, which has a vital role in glucose tolerance related to reductions in endotoxins and proinflammatory cytokines and permeability of the intestine [98].

Studies have reported that in experimental models of high-fat-diet-induced diabetes and the administration of streptozotocin, *Lactobacillus* spp., *Bifidobacterium animalis, Bifidobacterium animalis* subsp. *lactis, B. adolescentis*, and *B.* *breve* improved insulin sensitivity, regulated glucose metabolism, and reduced proinflammatory cytokines, liver lipids, and body weight [99,100]. Indeed, *B. animalis* decreases body weight, hyperglycemia, TC levels, lipopolysaccharides, TNF-α, the enzyme activity of the necrosis markers ALT and AST, as well as oxidative stress; in addition, it has an antidiabetic effect by regulation of the RS/PI3K/AKT and Keap1/Nrf2 pathways. *Lactobacillus* species have improved glucose intolerance, hyperglycemia, hyperinsulinemia, dyslipidemia, and oxidative stress. *Akkermansia muciniphila* also decreased glucose-6-phosphatase to counteract fasting hyperglycemia, suggesting that it may decrease gluconeogenesis [3,69].

The main mechanisms identified for probiotics and prebiotics in managing type 2 diabetes are reduction of oxidative stress, modulation of proinflammatory cytokines and CRP, bioactive peptides and SCFA production, glycemic and lipidic regulation, and intestinal permeability improvement, besides the reduction of insulin resistance [101,102,103,104].

## 6. Mechanisms of Action

**Figure 1:**
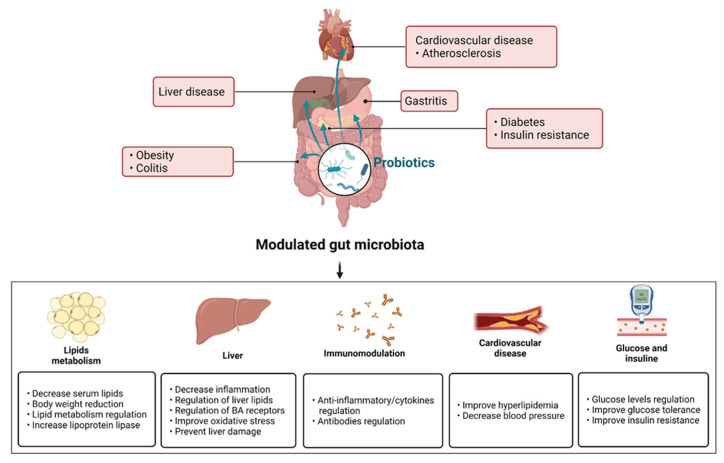
A balanced gut microbiota implies an increase in the abundance and diversity of beneficial species and a decrease in pathogenic bacteria. This leads to reduction in intestinal hyperpermeability and the prevention of infections. Prebiotics, probiotics, and synbiotics are related to hepatic, cardiovascular, gastrointestinal, and metabolic disorders; for example, liver injury, such as NAFLD and NASH; gastrointestinal diseases, such as gastritis and colitis; diseases related to lipid metabolism, including hyperlipidemia, obesity, and atherosclerosis; and diabetes. The administration of prebiotics, probiotics, and synbiotics confers various benefits. In lipid metabolism, serum lipid regulation has been reported, specifically of TC, VLDL-C, LDL-C, TG, and TBA; this regulation has been related to the low expression of SREBP-1c and CYP7A1, among other effects, in addition to the decrease in body weight and the positive regulation of PPAR-α, PPAR-γ, and lipoprotein lipase. Liver injury has been improved to reduce AST, ALT, AP, oxidative stress, and hepatic lipid accumulation. An immunomodulatory effect was observed, mainly anti-inflammatory, due to the decrease in proinflammatory cytokines (TNF-alpha, IL-6, IL-1β, and C-reactive protein) and the increased expression of anti-inflammatory cytokine IL-10 and the antibody IgA. Cardiovascular diseases have been improved for blood pressure regulation (systolic and diastolic) and serum levels decreased in hyperlipidemia. Finally, in diabetes, significant reductions in glucose serum levels, fasting serum glucose, glucose tolerance, and insulin resistance have been observed.

The beneficial effects of prebiotics, probiotics, synbiotics, paraprobiotics, and postbiotics in metabolic and hepatogastrointestinal disorders have been attributed to the mechanisms of action described in this section (Figure 1) [20,105,106].

### 6.1. Immunomodulation

Some probiotics show direct antimicrobial activity through the production of metabolites; others show non-immunological activity, such as competing with pathogenic microorganisms for nutrients, altering intestinal pH, increasing mucus production, or contributing to tissue repair, thus reducing the permeability of the intestinal mucosa [14]. However, several studies have shown that when there are deficiencies in probiotics, there are also deficiencies in the immune system. Probiotics can interact with the innate and adaptive immune system, contributing to the regulation of cell differentiation that is involved with immune tolerance, mainly contributing to the activation of dendritic cells, and therefore driving the differentiation of regulatory T cells; this implies a stimulus for Th1, Th2, and Th17 cells [107]. Probiotics have also been reported to have a favorable effect in cases of autoimmune and allergic responses, as they induce the production of anti-inflammatory cytokines, such as IL-4, IL-10, and TGF-β, promoting the proliferation of CD4+ T lymphocytes [15,35]. 

Dendritic cells regulate the immune system and influence the differentiation of Treg and Th17 and IgA production, resulting in the production of proinflammatory cytokines (IL-17, IL-23). Probiotics stimulate TLR activation and immune responses towards Th1/Th2 and Treg phenotypes. They also promote natural killer cell (NK) activity, induce T cell apoptosis, regulate the expression of anti-inflammatory cytokines (IL-10, TGF-β), and reduce proinflammatory cytokines (TGF-α, interferon (IFN)-γ) [108,109].

On the other hand, prebiotics can modulate the immune response directly or indirectly by balancing the gut microbiota or by being metabolized by microorganisms to produce microbial compounds, such as SCFA. Prebiotics such as inulin can modulate IgG and IgA antibodies and the expression of genes for cytokine production (for example, FOS/inulin and oligofructose/inulin) and can also reduce pathogens; for example, some oligosaccharides have anti-adherent properties against enteropathogenic bacteria and enterotoxins [110,111,112,113].

### 6.2. Lipid Homeostasis

Concerning the role of microbiota in regulating cholesterol homeostasis, it has been found that when a high amount of lipids is present, there is a loss in the diversity and richness of the intestinal microbiota. The underlying molecular mechanisms involve a decrease in the signaling of the nuclear receptors PPAR, FXR, and TGR-5 [114,115]. The PPAR nuclear transcription factors have a crucial role in protein, carbohydrate, and lipid homeostasis; they stimulate lipoprotein lipase expression, leading to a decrease in triglycerides and an increase in HDL-C, and they also regulate β-oxidation [116,117]. BA activates FXR and TGR-5 receptors. FXR keeps BA at low levels in hepatocytes to prevent liver injury, mainly cholestasis, and toxicity by their accumulation; also, FXR inhibits the transcription of CYP7A1 and lipogenesis mediated by SREBP-1c and induces PPAR receptors [118,119]. TGR-5 displays immunomodulatory activity by inhibiting the secretion of proinflammatory cytokines, a process in which the nuclear factor NF-κB is involved and related to NO synthesis [120,121]. Otherwise, liver X receptors (LXR) promote lipogenesis by upregulating the expression of acetyl-CoA carboxylase, fatty acid synthase, stearoyl-CoA desaturase-1 (SCD-1), and SREBP-1c; the latter is a transcription factor that controls the expression of most genes involved in fatty acid biosynthesis. Therefore, LXR and SREBP-1c expression increases during metabolic disorders, and the activation or regulation of FXR, TGR-5, and PPAR modulates carbohydrate and lipid metabolism and inflammatory processes by binding to BA. For example, it has been demonstrated that fxr knockout mice had insulin resistance and hyperglycemia; meanwhile, FXR agonist GW4064 administration decreased serum glucose, increased liver glycogen, and improved insulin sensitivity. On the other hand, it has been observed in hypercholesterolemic animal models that activation of FXR decreases LDL-C and promotes reverse cholesterol transport by inducing hepatic expression of scavenger receptor B1 (SRB1) and induces apolipoprotein CII (ApoCII), which is a lipoprotein lipase activator [105,122,123]. 

### 6.3. Bile Acid Metabolism

BAs are metabolic regulators that control gut bacteria growth to regulate glucose and lipid homeostasis. If this metabolism is deregulated, this change causes dysbiosis, leading to obesity, diabetes, liver-related diseases, and other aforementioned diseases [124]. Their biotransformation implies a close relationship with the intestinal microbiota. Hepatocytes synthesize primary BAs from cholesterol; subsequently, they enter the gastrointestinal tract, where the microbiota modifies them to form secondary BAs [125]. 

About 5% of unabsorbed BAs enter the distal ileum, cecum, and colon; they undergo chemical diversification through three microbial pathways: deconjugation reactions, dehydrogenation, and dehydroxylation. Deconjugation occurs through bile salt hydrolases (BSHs) present in various Firmicutes, Bacteroidetes, and Actinobacteria species, specifically the genera *Clostridium*, *Bacteroides*, *Lactobacillus*, *Bifidobacterium*, and *Enterococcus* [126]. BSHs render nutrients by releasing amino acids that can be used as carbon/nitrogen sources and for energy generation through taurine as a terminal electron acceptor; also, they participate in the integration of cholesterol and bile components into bacterial membranes and provide a detoxification mechanism to decrease the inherent detergent properties of BAs. Three distinct microbial hydroxysteroid dehydrogenases (3α-, 7α-, and 12α-HSDH) are present in the microbiota and complete the oxidation and epimerization of specific hydroxyl groups in BAs. Most BAs are of microbial origin in the colon, and unconjugated BAs undergo dehydrogenation by a broad spectrum of bacteria. For instance, 7α-dehydroxylation of BAs can only be performed by anaerobic species *(Peptacetobacter hiranonis*, *Clostridium* *hylemonae*, *C. scindens,* and *Paeniclostridium sordellii)*. The elimination of the 7α-hydroxyl group of host-derived primary BAs requires multiple stages of intracellular enzymes; this ultimately leads to the formation of the secondary bile deoxycholic acid (DCA) from cholic acid (CA) and lithocholic acid (LCA), which can be modified into other secondary BAs, such as isoDCA and isoLCA. BAs differ in their activation of FXR (CDCA > DCA > LCA > CA); therefore, BA diversification by the microbiota could directly influence the ability of FXR to inhibit lipogenesis [125,127,128]. 

## 7. Conclusions

The intestinal microbiota plays an important role not only in the prevention of various diseases but also has been shown to improve a wide range of medical conditions worldwide, such as disorders of the gastrointestinal system, particularly problems related to inflammatory bowel disease, which can lead to more severe conditions, including some types of cancer. Moreover, its usefulness in metabolic disorders associated with cardiovascular system diseases has also been evidenced. Several authors attribute these benefits to various mechanisms of action of probiotics; among the best evaluated are the ability to modulate the immune response mainly through regulating the secretion of pro- and anti-inflammatory cytokines as well as lipid and carbohydrate metabolism regulation. Additionally, probiotics play a vital role in the host metabolism/transformation of bile salts, so they mediate various pathways of activation and the expression of related genes, thus leading to significant changes in such diseases. It has been shown that keeping the intestinal microbiota balanced can prevent the development of many diseases, including some which have not been addressed in the present work, although supported by several studies relating to diseases of the central nervous system, autoimmune disease, infections, etc. When dysbiosis develops, prebiotic, probiotic, or synbiotic consumption contributes to increasing the populations of beneficial species and, consequently, improves or prevents metabolic disorders and hepatogastrointestinal diseases, which are also correlated. A wide variety of these products and even products that contain paraprobitics are available, and, together with an adequate diet, favor the reestablishment of the intestinal microbiota. However, it is necessary to carry out more studies that validate the mechanisms of action related to the effects of probiotics and prebiotics that can also be consumed in many foods already incorporated into the diet.

## Figures and Tables

**Table 1:** Effects of prebiotics, probiotics, and synbiotics in liver disease, colitis, gastritis, hyperlipidemia, obesity, and diabetes.

Liver Injury			
Model	Prebiotic or Probiotic Species/Treatments	Effect	Reference
C57BL/6 mice. Obesity and NAFLD induced by HFD	Sapogenins	↓TNF-α ↓SREBP-1c ↑PPAR-α ↑*A. muciniphila*	[34]
Wistar rats. Fibrosis induced by TAA	*Lacticaseibacillus paracasei, L. casei, Weissella confuse*	↓TNF-α ↓TGF-β ↓α-SMA ↓ALT, AST, AP	[55]
Sprague Dawley rats. Acute liver injury induced by D-galactosamine	*Lacticaseibacillus casei* Shirota	↓GGTP ↓TBA ↓G-CSF ↓RANTES ↓IL-5 ↑IL-10	[56]
Patients with NAFLD	Synbiotic (Oligofructose and *Lacticaseibacillus casei, L. rhamnosus, Lactobacillus acidophilus, Bifidobacterium longum, B. breve*)	↓ALT, AST, GGTP, AP ↓TG, TC, LDL	[57]
Patients with NAFLD	*Bacillus coagulans* and *inuline*	↓ALT, GGTP ↓TNF-α ↓NF-κB	[58]
Patients with NASH	Synbiotic (*Bifidobacterium longum* and FOS)	↓AST ↓TNF-α ↓LDL-C ↓CRP ↓Serum endotoxin Insulin resistance, steatosis, and NASH activity index were improved	[59]
Wistar rats. Acute liver injury induced by acetaminophen	*Bacillus licheniformis, B. indicus, B. subtilis, Shouchella clausii, Weizmannia coagulans*	↓AST, ALT ↓TNF-α, ↓IL-1β ↓Necrosis Antioxidant capacity	[60]
Zucker-Lepr *^fa/fa^* rats (NAFLD).	*Bifidobacterium breve, Lacticaseibacillus paracasei, L. rhamnosus*	↓MDA, MPO ↓Hepatic lipids ↓TNF-α, IL-6, LPS ↑IgA	[61]
**Colitis**			
C57BL/6 mice. Colitis-associated cancer induced by azoxymethane and DSS	*Lactobacillus delbrueckii* subsp. *bulgaricus*	Fewer and smaller tumors than the control Attenuation of intestinal inflammation ↑INF-γ ↓TNF-α ↓IL-1β, IL-23, IL-17, IL-6	[62]
BALB/c mice. Colitis induced by DSS	*Lacticaseibacillus rhamnosus* and/or *inuline*	↑ Abundance and diversity of gut microbiota ↑Hemoglobin content ↓MPO ↓TNF-α ↓IL-1β, IL-6 ↑IL-10	[63]
C57BL/6 mice. Colitis induced by DSS	FOS Synbiotic (FOS + Limosilactobacillus reuteri, *L. fermentum, Lacticaseibacillus paracasei, L. rhamnosus, Bifidobacterium animalis* subsp. *lactis, B. breve, and Streptococcus salivarius* subsp. *thermophilus*)	↑Abundance and diversity of gut microbiota ↓Abundance of potentially harmful bacteria ↓TNF-α ↓IL-1β, IL-6 ↑IL-10 Regulated expression of T-bet	[64]
C57BL/6 mice. Colitis induced by DSS	Synbiotic (*Lactobacillus acidophilus, Lacticaseibacillus rhamnosus, Bifidobacterium animalis* subsp. *lactis* + *inuline*)	Attenuation of intestinal inflammation Balance of intestinal microbiota	[65]
C57BL/6J mice. Colitis induced by DSS	Prebiotic (FOS + XOS) Probiotic (*Lactobacillus acidophilus*, *Bifidobacterium animalis* subsp. *lactis*, *Limosilactobacillus reuteri*, *Lacticaseibacillus rhamnosus*, *Streptococcus salivarius* subsp. *thermophilus*) Synbiotic (Prebiotic + Probiotic)	↓TNF-α ↓IL-1β, IL-6 ↑IL-10 ↑Firmicutes ↓STAT3	[66]
C57BL/6 mice. Colitis induced by DSS	Probiotic (*Lactobacillus acidophilus, L. helveticus, Lacticaseibacillus casei, L. rhamnosus, L. paracasei, Limosilactobacillus fermentum, Streptococcus salivarius* subsp. *thermophilus, Bifidobacterium longum, B. breve, B. bifidum,* *Lactiplantibacillus plantarum, and Ligilactobacillus salivarius*) Prebiotic (Chicory Fiber)	↓TNF-α ↓IL-1β, IL-6 ↓IL-17 ↑IL-10 ↑Foxp3 ↓α-SMA ↓MCP-1 in HT-29 cells	[67]
**Diabetes/Obesity/Hyperlipidemia**			
Wistar rats. Diabetes (type 2) induced by HFD and STZ	*Pediococcus acidilactici,* *Lactiplantibacillus plantarum,* *Lacticaseibacillus rhamnosus*	Glucose levels restored Prevention against hyperlipidemia Improved glucose tolerance	[68]
Sprague-Dawley rats. Diabetes (type 2) induced by HFD and STZ	*Bifidobacterium animalis*	↓HbA_1C_, fasting blood glucose Glucose tolerance and insulin resistance improved ↓TC, LDL-C ↓LPS ↓TNF-α ↑IL-10 ↓ALT and AST IRS-2/PI3K/Akt-2 signal Pathway improved ↓PEPCK, G6Pase	[69]
Diabetic (type 2) female patients.	Enriched chicory inulin supplementation	↓Fasting serum glucose ↓HbA_1C_ ↓ALT, AST, AP Reductions in systolic and diastolic blood pressure	[70]
Diabetic (type 2) patients.	Synbiotic (*Lactobacillus, Bifidobacterium, Lactococcus, Propionibacterium, Acetobacter*, and Omega-3)	Insulin resistance improved ↓IL-1β, IL-6, IL-8 ↓TNF-α	[71]
C57BL/6 JRj mice. Obesity-induced by HFD	*Bifidobacterium longum* *B. animalis* subsp. *lactis* and *Lactobacillus gasseri*	↑PPAR-γ ↑Lipoprotein lipase ↑GLP-1 ↑IL-10 ↓Lipids accumulation in adipocytes Restored gut barrier Limited body weight gain	[72]
Wistar rats. Obesity and insulin resistance induced by HFD	Prebiotic (XOS) Probiotic (*Lacticaseibacillus paracasei*) Synbiotic (Prebiotic + Probiotic)	↓Plasma insulin ↓TC ↓LDL-C Insulin resistance improved Bodyweight and visceral fat weight reduced ↓BP and MAP ↓Oxidative stress	[73]
Sprague Dawley rats. Hyperlipidemia induced by HFD	*Lacticaseibacillus rhamnosus* LV108, *Lactobacillus casei* grx12, and *Limosilactobacillus fermentum* grx08	↓TC, VLDL-C, LDL-C ↓TG ↓TBA ↓SREBP-1c ↓ChREBP ↑PPAR-α ↓CYP7A1	[74]

Please see the glossary for abbreviations; ↓ (decrease), ↑ (increase).

## Data Availability

Not applicable.

## Abbreviations

ALT	Alanine aminotransferase
AP	Alkaline phosphatase
AST	Aspartate aminotransferase
BA(s)	Bile acid(s)
BP	Blood pressure
BSHs	Bile salt hydrolases
CA	Cholic acid
CRP	C-reactive protein
DCA	Deoxycholic acid
DSS	Dextran sulfate sodium
FOS	Fructooligosaccharide
FXR	Farnesoid X receptor
G6Pase	Glucose-6-phosphatase
GLP-1	Glucagon-like peptide 1
GOS	Galactooligosaccharides
GSH-Px	Glutathione peroxidase
H_2_O_2_	Hydrogen peroxide
HCC	Hepatocellular carcinoma
HDL-C	High-density lipoprotein cholesterol
HFD	High-fat diet
HSDH	Hydroxysteroid dehydrogenases
IBD	Inflammatory bowel diseases
IFN-γ	Interferon-γ
IgA	Immunoglobulin A
IgG	Immunoglobulin G
IL	Interleukin(s)
ISAPP	International Scientific Association of Probiotics and Prebiotics
KO	Knockout model
LCA	Lithocholic acid
LDL-C	Low-density lipoprotein cholesterol
LPS	Lipopolysaccharides
LXR	Liver X receptor
MAP	Mean arterial pressure
MDA	Malondialdehyde
MetS	Metabolic syndrome
MPO	Myeloperoxidase
NAFLD	Non-alcoholic fatty liver disease
NASH	Non-alcoholic steatohepatitis
NF-κB	Nuclear factor kappa-light-chain-enhancer of activated B cells
NK	Natural killer
NO	Nitric oxide
PPAR	Peroxisome proliferator-activated receptor
SCD-1	Stearoyl-CoA desaturase-1
SCFA	Short-chain fatty acids
SOD	Superoxide dismutase
SREBP-1c	Sterol regulatory element-binding protein-1c
STZ	Streptozotocin
TAA	Thioacetamide
TBA	Total bile acids
TC	Total cholesterol
TG	Triglycerides
TGF-β	Transforming growth factor-β
TGR-5	Takeda G-protein Receptor-5
TLR	Toll-like receptors
TMA	Trimethylamine
TMAO	Trimethylamine N-oxide
TNF-α	Tumour Necrosis Factor-α
VLDL-C	Very low-density lipoprotein cholesterol
XOS	Xylo-oligosaccharide

## Appendix A

All the scientific genera and species were updated and validated according to the International Code of Nomenclature of Prokaryotes, Parker C.T.; Tindall B.J.; Garrity G.M. International Journal of Systematic and Evolutionary Microbiology, 2019, 69, S1-S111; also, the https://www.ezbiocloud.net/ (accessed on 5 June 2022) database was consulted.

## References

[1] Coșoreanu A., Rusu E., Rusu F., Băleanu M., Cîrstea C., Marinescu M., Radulian G. (2021). Probiotics and prebiotics in the prevention of gastrointestinal adverse reactions related to diabetes mellitus. Farmacia.

[2] Corb Aron R.A., Abid A., Vesa C.M., Nechifor A.C., Behl T., Ghitea T.C., Munteanu M.A., Fratila O., Andronie-Cioara F.L., Toma M.M. (2021). Recognizing the Benefits of Pre-/Probiotics in Metabolic Syndrome and Type 2 Diabetes Mellitus Considering the Influence of *Akkermansia muciniphila* as a Key Gut Bacterium. Microorganisms.

[3] He M., Shi B. (2017). Gut microbiota as a potential target of metabolic syndrome: The role of probiotics and prebiotics. Cell Biosci..

[4] Fernández-Martínez E., Lira-Islas I.G., Cariño-Cortés R., Soria-Jasso L.E., Pérez-Hernández E., Pérez-Hernández N. (2019). Dietary chia seeds (*Salvia hispanica*) improve acute dyslipidemia and steatohepatitis in rats. J. Food Biochem..

[5] Cho M.S., Kim S.Y., Suk K.T., Kim B.Y. (2018). Modulation of gut microbiome in non-alcoholic fatty liver disease: Pro-, pre-, syn-, and antibiotics. J. Microbiol..

[6] Castillo V., Figueroa F., González-Pizarro K., Jopia P., Ibacache-Quiroga C. (2021). Probiotics and Prebiotics as a Strategy for Non-Alcoholic Fatty Liver Disease, a Narrative Review. Foods.

[7] Liu L., Li P., Liu Y., Zhang Y. (2019). Efficacy of Probiotics and Synbiotics in Patients with Nonalcoholic Fatty Liver Disease: A Meta-Analysis. Dig. Dis. Sci..

[8] Jandhyala S.M., Talukdar R., Subramanyam C., Vuyyuru H., Sasikala M., Reddy D.N. (2015). Role of the normal gut microbiota. World J. Gastroenterol..

[9] Thursby E., Juge N. (2017). Introduction to the human gut microbiota. Biochem. J..

[10] Huang K., Wu L., Yang Y. (2021). Gut microbiota: An emerging biological diagnostic and treatment approach for gastrointestinal diseases. JGH Open.

[11] Quigley E.M.M. (2019). Prebiotics and probiotics in digestive health. Clin. Gastroenterol. Hepatol..

[12] Sun L., Pang Y., Wang X., Wu Q., Liu H., Liu B., Liu G., Ye M., Kong W., Jiang C. (2019). Ablation of gut microbiota alleviates obesity-induced hepatic steatosis and glucose intolerance by modulating bile acid metabolism in hamsters. Acta Pharm. Sin. B.

[13] Kori M., Daugule I., Urbonas V. (2018). *Helicobacter pylori* and some aspects of gut microbiota in children. Helicobacter.

[14] Akutko K., Stawarski A. (2021). Probiotics, Prebiotics and Synbiotics in Inflammatory Bowel Diseases. J. Clin. Med..

[15] Dargahi N., Johnson J., Donkor O., Vasiljevic T., Apostolopoulos V. (2019). Immunomodulatory effects of probiotics: Can they be used to treat allergies and autoimmune diseases?. Maturitas.

[16] Estrada-Godina A.R., Cruz-Guerrero A.E., Lappe P., Ulloa M., García-Garibay M., Gómez-Ruiz L. (2001). Isolation and identification of killer yeasts from *Agave* sap (mead) and pulque. World J. Microbiol. Biotechnol..

[17] Gibson G.R., Hutkins R., Sanders M.E., Prescott S.L., Reimer R.A., Salminen S.J., Scott K., Stanton C., Swanson K.S., Cani P.D. (2017). Expert consensus document: The International Scientific Association for Probiotics and Prebiotics (ISAPP) consensus statement on the definition and scope of prebiotics. Nat. Rev. Gastroenterol. Hepatol..

[18] Bindels L.B., Delzenne N.M., Cani P.D., Walter J. (2015). Towards a more comprehensive concept for prebiotics. Nat. Rev. Gastroenterol. Hepatol..

[19] Van Loo J. (2004). The specificity of the interaction with intestinal bacterial fermentation by prebiotics determines their physiological efficacy. Nutr. Res. Rev..

[20] Martyniak A., Medyńska-Przęczek A., Wędrychowicz A., Skoczeń S., Tomasik P.J. (2021). Prebiotics, Probiotics, Synbiotics, Paraprobiotics and Postbiotic Compounds in IBD. Biomolecules.

[21] Levy M., Kolodziejczyk A.A., Thaiss C.A., Elinav E. (2017). Dysbiosis and the immune system. Nat. Rev. Immunol..

[22] Hill C., Guarner F., Reid G., Gibson G.R., Merenstein D.J., Pot B., Morelli L., Canani R.B., Flint H.J., Salminen S. (2014). Expert consensus document. The International Scientific Association for Probiotics and Prebiotics consensus statement on the scope and appropriate use of the term probiotic. Nat. Rev. Gastroenterol. Hepatol..

[23] Soriano G., Sánchez E., Guarner C. (2013). Probiotics in liver diseases. Nutr. Hosp..

[24] Schwenger K.J., Clermont-Dejean N., Allard J.P. (2019). The role of the gut microbiome in chronic liver disease: The clinical evidence revised. JHEP Rep..

[25] Zendeboodi F., Khorshidian N., Mortazavian A.M., da Cruz A.G. (2020). Probiotic: Conceptualization from a new approach. Curr. Opin. Food Sci..

[26] Taverniti V., Guglielmetti S. (2011). The immunomodulatory properties of probiotic microorganisms beyond their viability (ghost probiotics: Proposal of paraprobiotic concept). Genes Nutr..

[27] Siciliano R.A., Reale A., Mazzeo M.F., Morandi S., Silvetti T., Brasca M. (2021). Paraprobiotics: A New Perspective for Functional Foods and Nutraceuticals. Nutrients.

[28] Tsilingiri K., Rescigno M. (2013). Postbiotics: What else?. Benef. Microbes.

[29] Weiss G.A., Hennet T. (2017). Mechanisms and consequences of intestinal dysbiosis. Cell. Mol. Life Sci..

[30] Lee N.Y., Suk K.T. (2021). The Role of the Gut Microbiome in Liver Cirrhosis Treatment. Int. J. Mol. Sci..

[31] Bull M.J., Plummer N.T. (2014). Part 1: The Human Gut Microbiome in Health and Disease. Integr. Med..

[32] Ray A., Dittel B.N. (2015). Interrelatedness between dysbiosis in the gut microbiota due to immunodeficiency and disease penetrance of colitis. Immunology.

[33] Ni J., Wu G.D., Albenberg L., Tomov V.T. (2017). Gut microbiota and IBD: Causation or correlation?. Nat. Rev. Gastroenterol. Hepatol..

[34] Leal-Díaz A.M., Noriega L.G., Torre-Villalvazo I., Torres N., Alemán-Escondrillas G., López-Romero P., Sánchez-Tapia M., Aguilar-López M., Furuzawa-Carballeda J., Velázquez-Villegas L.A. (2016). Aguamiel concentrate from *Agave salmiana* and its extracted saponins attenuated obesity and hepatic steatosis and increased *Akkermansia muciniphila* in C57BL6 mice. Sci. Rep..

[35] Torres-Maravilla E., Lenoir M., Mayorga-Reyes L., Allain T., Sokol H., Langella P., Sánchez-Pardo M.E., Bermúdez-Humarán L.G. (2016). Identification of novel anti-inflammatory probiotic strains isolated from pulque. Appl. Microbiol. Biotechnol..

[36] Philips C.A., Augustine P. (2022). Gut Barrier and Microbiota in Cirrhosis. J. Clin. Exp. Hepatol..

[37] Chikkerur J., Samanta A.K., Kolte A.P., Dhali A., Roy S. (2020). Production of Short Chain Fructo-oligosaccharides from Inulin of Chicory Root Using Fungal Endoinulinase. Appl. Biochem. Biotechnol..

[38] Altamirano-Barrera A., Uribe M., Chávez-Tapia N.C., Nuño-Lámbarri N. (2018). The role of the gut microbiota in the pathology and prevention of liver disease. J. Nutr. Biochem..

[39] Gupta H., Youn G.S., Shin M.J., Suk K.T. (2019). Role of Gut Microbiota in Hepatocarcinogenesis. Microorganisms.

[40] Jia B., Jeon C.O. (2019). Promotion and induction of liver cancer by gut microbiome-mediated modulation of bile acids. PLoS Pathog..

[41] Ming-mei L., Zhou Y., Zuo L., Nie D., Xiao-an L. (2020). Dietary fiber regulates intestinal flora and suppresses liver and systemic inflammation to alleviate liver fibrosis in mice. Nutrition.

[42] Koopman N., Molinaro A., Nieuwdorp M., Holleboom A.G. (2019). Review article: Can bugs be drugs? The potential of probiotics and prebiotics as treatment for non-alcoholic fatty liver disease. Aliment. Pharmacol. Ther..

[43] Nagashimada M., Honda M. (2021). Effect of Microbiome on Non-Alcoholic Fatty Liver Disease and the Role of Probiotics, Prebiotics, and Biogenics. Int. J. Mol. Sci..

[44] Astó E., Méndez I., Audivert S., Farran-Codina A., Espadaler J. (2019). The Efficacy of Probiotics, Prebiotic Inulin-Type Fructans, and Synbiotics in Human Ulcerative Colitis: A Systematic Review and Meta-Analysis. Nutrients.

[45] Zhang X.F., Guan X.X., Tang Y.J., Sun J.F., Wang X.K., Wang W.D., Fan J.M. (2021). Clinical effects and gut microbiota changes of using probiotics, prebiotics or synbiotics in inflammatory bowel disease: A systematic review and meta-analysis. Eur. J. Nutr..

[46] Schroeder B.O., Birchenough G.M.H., Ståhlman M., Arike L., Johansson M.E.V., Hansson G.C., Bäckhed F. (2018). Bifidobacteria or fiber protects against diet-induced microbiota-mediated colonic mucus deterioration. Cell Host Microbe.

[47] Akram W., Garud N., Joshi R. (2019). Role of inulin as prebiotics on inflammatory bowel disease. Drug Discov. Ther..

[48] Furrie E., Macfarlane S., Kennedy A., Cummings J.H., Walsh S.V., O’neil D.A., Macfarlane G.T. (2005). Synbiotic therapy (*Bifidobacterium longum*/Synergy 1) initiates resolution of inflammation in patients with active ulcerative colitis: A randomised controlled pilot trial. Gut.

[49] Lopez M., Li N., Kataria J., Russell M., Neu J. (2008). Live and ultraviolet-inactivated *Lactobacillus rhamnosus* GG decrease flagellin-induced interleukin-8 production in Caco-2 cells. J. Nutr..

[50] Chieng J.Y., Pan Y. (2022). The Role of Probiotics, Prebiotics and Synbiotics in Adult Gastrointestinal Health. Gastroenterol. Hepatol. Lett..

[51] Kelesidis T., Pothoulakis C. (2012). Efficacy and safety of the probiotic *Saccharomyces boulardii* for the prevention and therapy of gastrointestinal disorders. Ther. Adv. Gastroenterol..

[52] Song H.Y., Zhou L., Liu D.Y., Yao X.J., Li Y. (2018). What Roles Do Probiotics Play in the Eradication of *Helicobacter pylori*? Current Knowledge and Ongoing Research. Gastroenterol. Res. Pract..

[53] Ji J., Yang H. (2020). Using Probiotics as Supplementation for *Helicobacter pylori* Antibiotic Therapy. Int. J. Mol. Sci..

[54] Asefa Z. (2022). The Role of Probiotics in the Treatment of *Helicobacter Pylori*-Caused Gastritis. IJARP.

[55] Jantararussamee C., Rodniem S., Taweechotipatr M., Showpittapornchai U., Pradidarcheep W. (2021). Hepatoprotective effect of probiotic lactic acid bacteria on thioacetamide-induced liver fibrosis in rats. Probiot. Antimicrob. Proteins.

[56] Yan R., Wang K., Wang Q., Jiang H., Lu Y., Chen X., Zhang H., Su X., Du Y., Chen L. (2022). Probiotic *Lactobacillus casei* Shirota prevents acute liver injury by reshaping the gut microbiota to alleviate excessive inflammation and metabolic disorders. Microb. Biotechnol..

[57] Behrouz V., Aryaeian N., Zahedi M.J., Jazayeri S. (2020). Effects of probiotic and prebiotic supplementation on metabolic parameters, liver aminotransferases, and systemic inflammation in non-alcoholic fatty liver disease: A randomized clinical trial. J. Food Sci..

[58] Abhari K., Saadati S., Yari Z., Hosseini H., Hedayati M., Abhari S., Alavian S.M., Hekmatdoost A. (2020). The effects of *Bacillus coagulans* supplementation in patients with non-alcoholic fatty liver disease: A randomized, placebo-controlled, clinical trial. Clin. Nutr. ESPEN.

[59] Malaguarnera M., Vacante M., Antic T., Giordano M., Chisari G., Acquaviva R., Mastrojeni S., Malaguarnera G., Mistretta A., Li Volti G. (2012). *Bifidobacterium longum* with fructo-oligosaccharides in patients with non alcoholic steatohepatitis. Dig. Dis. Sci..

[60] Neag M.A., Catinean A., Muntean D.M., Pop M.R., Bocsan C.I., Botan E.C., Buzoianu A.D. (2020). Probiotic Bacillus Spores Protect against Acetaminophen Induced Acute Liver Injury in Rats. Nutrients.

[61] Fontana L., Plaza-Díaz J., Robles-Bolívar P., Valente-Godínez H., Sáez-Lara M.J., Abadía-Molina F., Gómez-Llorente C., Gil Á., Álvarez-Mercado A.I. (2021). *Bifidobacterium breve* CNCM I-4035 *Lactobacillus paracasei* CNCM I-4034 and *Lactobacillus rhamnosus* CNCM I-4036 Modulate Macrophage Gene Expression and Ameliorate Damage Markers in the Liver of Zucker-Lepr*^fa/fa^* Rats. Nutrients.

[62] Silveira D., Veronez L.C., Lopes-Júnior L.C., Anatriello E., Brunaldi M.O., Pereira-da-Silva G. (2020). *Lactobacillus bulgaricus* inhibits colitis-associated cancer via a negative regulation of intestinal inflammation in azoxymethane/dextran sodium sulfate model. World J. Gastroenterol..

[63] Liu Z., Liu F., Sun C., Wang W., Sun C., Gao D., Ma J., Hussain M.A., Xu C., Jiang Z. (2020). Study of the Alleviation Effects of a Combination of *Lactobacillus Rhamnosus* and Inulin on Mice with Colitis. Food Funct..

[64] Liao M., Zhang Y., Qiu Y., Wu Z., Zhong Z., Zeng X., Zeng Y., Xiong L., Yu W., Liu R. (2021). Fructooligosaccharide supplementation alleviated the pathological immune response and prevented the impairment of intestinal barrier in DSS-induced acute colitis mice. Food Funct..

[65] Wang Y.N., Meng X.C., Dong Y.F., Zhao X.H., Qian J.M., Wang H.Y., Li J.N. (2019). Effects of probiotics and prebiotics on intestinal microbiota in mice with acute colitis based on 16S rRNA gene sequencing. Chin. Med. J..

[66] Wong W.Y., Chan B.D., Leung T.-W., Chen M., Tai W.C.-S. (2022). Beneficial and anti-inflammatory effects of formulated prebiotics, probiotics, and synbiotics in normal and acute colitis mice. J. Funct. Foods.

[67] Park J.S., Choi J., Kwon J.Y., Jung K.A., Yang C.W., Park S.H., Cho M.L. (2018). A probiotic complex, rosavin, zinc, and prebiotics ameliorate intestinal inflammation in an acute colitis mouse model. J. Transl. Med..

[68] Cai T., Wu H., Qin J., Qiao J., Yang Y., Wu Y., Qiao D., Xu H., Cao Y. (2019). In vitro evaluation by PCA and AHP of potential antidiabetic properties of lactic acid bacteria isolated from traditional fermented food. LWT.

[69] Zhang J., Wang S., Zeng Z., Qin Y., Shen Q., Li P. (2020). Antidiabetic effects of *Bifidobacterium animalis* 01 through improving hepatic insulin sensitivity in type 2 diabetic rat model. J. Funct. Foods.

[70] Farhangi M.A., Javid A.Z., Dehghan P. (2016). The effect of enriched chicory inulin on liver enzymes, calcium homeostasis and hematological parameters in patients with type 2 diabetes mellitus: A randomized placebo-controlled trial. Prim. Care Diabetes.

[71] Kobyliak N., Falalyeyeva T., Mykhalchyshyn G., Molochek N., Savchuk O., Kyriienko D., Komisarenko I. (2020). Probiotic and omega-3 polyunsaturated fatty acids supplementation reduces insulin resistance, improves glycemia and obesity parameters in individuals with type 2 diabetes: A randomised controlled trial. Obes. Med..

[72] Alard J., Cudennec B., Boutillier D., Peucelle V., Descat A., Decoin R., Kuylle S., Jablaoui A., Rhimi M., Wolowczuk I. (2021). Multiple Selection Criteria for Probiotic Strains with High Potential for Obesity Management. Nutrients.

[73] Tunapong W., Apaijai N., Yasom S., Tanajak P., Wanchai K., Chunchai T., Kerdphoo S., Eaimworawuthikul S., Thiennimitr P., Pongchaidecha A. (2018). Chronic treatment with prebiotics, probiotics and synbiotics attenuated cardiac dysfunction by improving cardiac mitochondrial dysfunction in male obese insulin-resistant rats. Eur. J. Nutr..

[74] Wa Y., Yin B., He Y., Xi W., Huang Y., Wang C., Guo F., Gu R. (2019). Effects of Single Probiotic- and Combined Probiotic-Fermented Milk on Lipid Metabolism in Hyperlipidemic Rats. Front. Microbiol..

[75] Arora T., Singh S., Sharma R.K. (2013). Probiotics: Interaction with gut microbiome and anti-obesity potential. Nutrition.

[76] Lim P.S., Loke C.F., Ho Y.W., Hui Yin T. (2020). Cholesterol homeostasis associated with probiotic supplementation in vivo. J. Appl. Microbiol..

[77] Kovatcheva-Datchary P., Arora T. (2013). Nutrition, the gut microbiome and the metabolic syndrome. Best Pract. Res. Clin. Gastroenterol..

[78] Cani P.D., Amar J., Iglesias M.A., Poggi M., Knauf C., Bastelica D., Neyrinck A.M., Fava F., Tuohy K.M., Chabo C. (2007). Metabolic endotoxemia initiates obesity and insulin resistance. Diabetes.

[79] Kim B., Choi H.N., Yim J.E. (2019). Effect of Diet on the Gut Microbiota Associated with Obesity. J. Obes. Metab. Syndr..

[80] Cerdó T., García-Santos J.A., Bermúdez M.G., Campoy C. (2019). The Role of Probiotics and Prebiotics in the Prevention and Treatment of Obesity. Nutrients.

[81] Choque-Delgado G.T., Tamashiro W.M.D.S.C. (2018). Role of prebiotics in regulation of microbiota and prevention of obesity. Food Res. Int..

[82] Włodarczyk M., Śliżewska K. (2021). Obesity as the 21st Century’s major disease: The role of probiotics and prebiotics in prevention and treatment. Food Biosci..

[83] Vallianou N., Stratigou T., Christodoulatos G.S., Tsigalou C., Dalamaga M. (2020). Probiotics, Prebiotics, Synbiotics, Postbiotics, and Obesity: Current Evidence, Controversies, and Perspectives. Curr. Obes. Rep..

[84] Aoun A., Darwish F., Hamod N. (2020). The Influence of the Gut Microbiome on Obesity in Adults and the Role of Probiotics, Prebiotics, and Synbiotics for Weight Loss. Prev. Nutr. Food Sci..

[85] Yoo J.Y., Sniffen S., McGill Percy K.C., Pallaval V.B., Chidipi B. (2022). Gut Dysbiosis and Immune System in Atherosclerotic Cardiovascular Disease (ACVD). Microorganisms.

[86] Ma J., Li H. (2018). The Role of Gut Microbiota in Atherosclerosis and Hypertension. Front. Pharmacol..

[87] Mantziaris V., Kolios G. (2019). Gut Microbiota, Atherosclerosis, and Therapeutic Targets. Crit. Pathw. Cardiol..

[88] Verhaar B.J.H., Prodan A., Nieuwdorp M., Muller M. (2020). Gut Microbiota in Hypertension and Atherosclerosis: A Review. Nutrients.

[89] Pieczynska M.D., Yang Y., Petrykowski S., Horbanczuk O.K., Atanasov A.G., Horbanczuk J.O. (2020). Gut Microbiota and Its Metabolites in Atherosclerosis Development. Molecules.

[90] Duttaroy A.K. (2021). Role of Gut Microbiota and Their Metabolites on Atherosclerosis, Hypertension and Human Blood Platelet Function: A Review. Nutrients.

[91] Vourakis M., Mayer G., Rousseau G. (2021). The Role of Gut Microbiota on Cholesterol Metabolism in Atherosclerosis. Int. J. Mol. Sci..

[92] Oniszczuk A., Oniszczuk T., Gancarz M., Szymańska J. (2021). Role of Gut Microbiota, Probiotics and Prebiotics in the Cardiovascular Diseases. Molecules.

[93] Olas B. (2020). Probiotics, Prebiotics and Synbiotics—A Promising Strategy in Prevention and Treatment of Cardiovascular Diseases?. Int. J. Mol. Sci..

[94] Hassan A., Din A.U., Zhu Y., Zhang K., Li T., Wang Y., Luo Y., Wang G. (2019). Updates in understanding the hypocholesterolemia effect of probiotics on atherosclerosis. Appl. Microbiol. Biotechnol..

[95] Cabello-Olmo M., Araña M., Urtasun R., Encio I.J., Barajas M. (2021). Role of Postbiotics in Diabetes Mellitus: Current Knowledge and Future Perspectives. Foods.

[96] Mishra S., Wang S., Nagpal R., Miller B., Singh R., Taraphder S., Yadav H. (2019). Probiotics and Prebiotics for the Amelioration of Type 1 Diabetes: Present and Future Perspectives. Microorganisms.

[97] Alagiakrishnan K., Halverson T. (2021). Holistic perspective of the role of gut microbes in diabetes mellitus and its management. World J. Diabetes.

[98] Vyas N., Nair S., Rao M., Miraj S.S., Bagchi D. (2019). Chapter 29 -Childhood Obesity and Diabetes: Role of Probiotics and Prebiotics. Global Perspectives on Childhood Obesity.

[99] Chen J., Wang R., Li X.F., Wang R.L. (2012). *Bifidobacterium adolescentis* supplementation ameliorates visceral fat accumulation and insulin sensitivity in an experimental model of the metabolic syndrome. Br. J. Nutr..

[100] Rouxinol-Dias A.L., Pinto A.R., Janeiro C., Rodrigues D., Moreira M., Dias J., Pereira P. (2016). Probiotics for the control of obesity—Its effect on weight change. Porto Biomed. J..

[101] Zepeda-Hernández A., Garcia-Amezquita L.E., Requena T., García-Cayuela T. (2021). Probiotics, prebiotics, and synbiotics added to dairy products: Uses and applications to manage type 2 diabetes. Food Res. Int..

[102] Razmpoosh E., Javadi M., Ejtahed H.-S., Mirmiran P. (2016). Probiotics as beneficial agents in the management of diabetes mellitus: A systematic review. Diabetes Metab. Res. Rev..

[103] Ardeshirlarijani E., Tabatabaei-Malazy O., Mohseni S., Qorbani M., Larijani B., Baradar Jalili R. (2019). Effect of probiotics supplementation on glucose and oxidative stress in type 2 diabetes mellitus: A meta-analysis of randomized trials. Daru.

[104] Liang T., Wu L., Xi Y., Li Y., Xie X., Fan C., Yang L., Yang S., Chen X., Zhang J. (2021). Probiotics supplementation improves hyperglycemia, hypercholesterolemia, and hypertension in type 2 diabetes mellitus: An update of meta-analysis. Crit. Rev. Food Sci. Nutr..

[105] Chen Z., Chen H., Zhang Z., Ding P., Yan X., Li Y., Zhang S., Gu Q., Zhou H., Xu J. (2020). Discovery of novel liver X receptor inverse agonists as lipogenesis inhibitors. Eur. J. Med. Chem..

[106] Ridlon J.M., Kang D.J., Hylemon P.B., Bajaj J.S. (2014). Bile acids and the gut microbiome. Curr. Opin. Gastroenterol..

[107] Dargahi N., Johnson J., Donkor O., Vasiljevic T., Apostolopoulos V. (2018). Immunomodulatory effects of *Streptococcus thermophilus* on U937 monocyte cell cultures. J. Funct. Foods.

[108] Abraham B.P., Quigley E. (2017). Probiotics in Inflammatory Bowel Disease. Gastroenterol. Clin. N. Am..

[109] Ghavami S.B., Yadegar A., Aghdaei H.A., Sorrentino D., Farmani M., Mir A.S., Azimirad M., Balaii H., Shahrokh S., Zali M.R. (2020). Immunomodulation and Generation of Tolerogenic Dendritic Cells by Probiotic Bacteria in Patients with Inflammatory Bowel Disease. Int. J. Mol. Sci..

[110] Yahfoufi N., Mallet J., Graham E., Matar C. (2018). Role of probiotics and prebiotics in immunomodulation. Curr. Opin. Food Sci..

[111] Vinolo M.A.R., Rodrigues H.G., Nachbar R.T., Curi R. (2011). Regulation of Inflammation by Short Chain Fatty Acids. Nutrients.

[112] Vogt L., Meyer D., Pullens G., Faas M., Smelt M., Venema K., Ramasamy U., Schols H.A., De Vos P. (2015). Immunological properties of inulin-type fructans. Crit. Rev. Food Sci. Nutr..

[113] Herfel T.M., Jacobi S.K., Lin X., Fellner V., Walker D.C., Jouni Z.E., Odle J. (2011). Polydextrose enrichment of infant formula demonstrates prebiotic characteristics by altering intestinal microbiota, organic acid concentrations, and cytokine expression in suckling piglets. J. Nutr..

[114] Khovidhunkit W., Kim M.S., Memon R.A., Shigenaga J.K., Moser A.H., Feingold K.R., Grunfeld C. (2004). Effects of infection and inflammation on lipid and lipoprotein metabolism: Mechanisms and consequences to the host. J. Lipid Res..

[115] Villette R., Pukar K.C., Beliard S., Salas Tapia M.F., Rainteau D., Guerin M., Lesnik P. (2020). Unraveling host-gut microbiota dialogue and its impact on cholesterol levels. Front. Pharmacol..

[116] Shen Y., Su Y., Silva F.J., Weller A.H., Sostre-Colón J., Titchenell P.M., Steger D.J., Seale P., Soccio R.E. (2020). Shared PPARα/γ target genes regulate brown adipocyte thermogenic function. Cell Rep..

[117] Pawlak M., Lefebvre P., Staels B. (2015). Molecular mechanism of PPARα action and its impact on lipid metabolism, inflammation and fibrosis in non-alcoholic fatty liver disease. J. Hepatol..

[118] Kumari A., Pal Pathak D., Asthana S. (2020). Bile acids mediated potential functional interaction between FXR and FATP5 in the regulation of lipid metabolism. Int. J. Biol. Sci..

[119] Zhou H., Zhou S.Y., Gillilland M., Li J.Y., Lee A., Gao J., Zhang G., Xu X., Owyang C. (2020). Bile acid toxicity in Paneth cells contributes to gut dysbiosis induced by high-fat feeding. JCI Insight.

[120] Chiang J., Ferrell J.M. (2020). Bile acid receptors FXR and TGR5 signaling in fatty liver diseases and therapy. Am. J. Physiol. Gastrointest. Liver Physiol..

[121] Kida T., Tsubosaka Y., Hori M., Ozaki H., Murata T. (2013). Bile acid receptor TGR5 agonism induces NO production and reduces monocyte adhesion in vascular endothelial cells. Arterioscler. Thromb. Vasc. Biol..

[122] Linden A.G., Li S., Choi H.Y., Fang F., Fukasawa M., Uyeda K., Hammer R.E., Horton J.D., Engelking L.J., Liang G. (2018). Interplay between ChREBP and SREBP-1c coordinates postprandial glycolysis and lipogenesis in livers of mice. J. Lipid Res..

[123] Li T., Chiang J.Y.L. (2012). Bile Acid signaling in liver metabolism and diseases. J. Lipids.

[124] Chiang J.Y.L., Fiorucci S. (2021). Bile acid metabolism and bile acid receptor signaling in metabolic diseases and therapy. Liver Res..

[125] Winston J.A., Theriot C.M. (2019). Diversification of host bile acids by members of the gut microbiota. Gut Microbes.

[126] Yamada S., Takashina Y., Watanabe M., Nagamine R., Saito Y., Kamada N., Saito H. (2018). Bile acid metabolism regulated by the gut microbiota promotes non-alcoholic steatohepatitis-associated hepatocellular carcinoma in mice. Oncotarget.

[127] Li T., Chiang J. (2020). Bile acid-based therapies for non-alcoholic steatohepatitis and alcoholic liver disease. Hepatobiliary Surg. Nutr..

[128] Chiang J.Y.L., Ferrel J.M. (2022). Discovery of farnesoid X receptor and its role in bile acid metabolism. Mol. Cell. Endocrinol..

